# Genome Maintenance Proteins Modulate Autoimmunity Mediated Primed Adaptation by the *Escherichia coli* Type I-E CRISPR-Cas System

**DOI:** 10.3390/genes10110872

**Published:** 2019-10-31

**Authors:** Elena Kurilovich, Anna Shiriaeva, Anastasia Metlitskaya, Natalia Morozova, Ivana Ivancic-Bace, Konstantin Severinov, Ekaterina Savitskaya

**Affiliations:** 1 Center of Life Sciences, Skolkovo Institute of Science and Technology, Moscow 143028, Russia; elena.kurilovich@skoltech.ru (E.K.); annabiologic@gmail.com (A.S.); natusmorozovna@gmail.com (N.M.); 2 Waksman Institute, Rutgers, the State University of New Jersey, Piscataway, NJ 08854, USA; 3 Institute of Molecular Genetics, Russian Academy of Sciences, Moscow 123182, Russia; azmetlitskaya@gmail.com; 4 Peter the Great St Petersburg State Polytechnic University, St Petersburg 195251, Russia; 5 Department of Biology, Faculty of Science, University of Zagreb, Horvatovac 102a, 10000 Zagreb, Croatia; ivanai@irb.hr; 6 Center for Precision Genome Editing and Genetic Technologies for Biomedicine, Institute of Gene Biology, Russian Academy of Sciences, 34/5 Vavilov St., Moscow 119334, Russia

**Keywords:** CRISPR-Cas, *Escherichia coli*, primed adaptation, DNA repair

## Abstract

Bacteria and archaea use CRISPR-Cas adaptive immunity systems to interfere with viruses, plasmids, and other mobile genetic elements. During the process of adaptation, CRISPR-Cas systems acquire immunity by incorporating short fragments of invaders’ genomes into CRISPR arrays. The acquisition of fragments of host genomes leads to autoimmunity and may drive chromosomal rearrangements, negative cell selection, and influence bacterial evolution. In this study, we investigated the role of proteins involved in genome stability maintenance in spacer acquisition by the *Escherichia coli* type I-E CRISPR-Cas system targeting its own genome. We show here, that the deletion of *recJ* decreases adaptation efficiency and affects accuracy of spacers incorporation into CRISPR array. Primed adaptation efficiency is also dramatically inhibited in double mutants lacking *recB* and *sbcD* but not in single mutants suggesting independent involvement and redundancy of RecBCD and SbcCD pathways in spacer acquisition. While the presence of at least one of two complexes is crucial for efficient primed adaptation, RecBCD and SbcCD affect the pattern of acquired spacers. Overall, our data suggest distinct roles of the RecBCD and SbcCD complexes and of RecJ in spacer precursor selection and insertion into CRISPR array and highlight the functional interplay between CRISPR-Cas systems and host genome maintenance mechanisms.

## 1. Introduction

Arrays of clustered regularly interspaced short palindromic repeats (CRISPR) separated by variable spacers and *cas* (CRISPR-associated) genes constitute CRISPR-Cas adaptive immunity systems used by a wide range of bacteria and archaea to defend against invading DNA or RNA [1,2,3]. When targeting the host genome, CRISPR-Cas systems induce chromosomal rearrangements and/or cell death and in this way may drive bacterial evolution [4,5,6]. Induction of self-targeting by CRISPR-Cas also has been proposed as a promising approach for specific elimination of bacterial cells [7,8].

The Cas effector complexes consist of Cas protein(s) and crRNA, which is a product of CRISPR array transcription and processing. The effector complex recognizes and channels for degradation nucleic acids based on complementarity of a crRNA spacer and a region of a target called “protospacer” [9]. Target protospacer recognition by most CRISPR-Cas systems depends on a short *p*rotospacer *a*djacent *m*otif (PAM) [10,11]. Fragments of DNA inside the cell, including those of bacteriophage genomes and plasmids as well as host DNA, may serve as “prespacers” and be channeled for integration into CRISPR array as new spacers in a process of CRISPR adaptation [12]. The specificity of the adaptation process to PAM-containing DNA fragments in CRISPR-Cas systems that rely on PAM ensures that acquired spacers generate crRNAs capable of interference.

The most common CRISPR-Cas system of *Escherichia coli* belongs to type I-E and comprises a multisubunit target recognition complex Cascade-crRNA, a Cas3 nuclease-helicase that destroys Cascade-bound targets, and the Cas1-Cas2 adaptation complex [2,13]. The consensus PAM in this system is an AAG trinucleotide located upstream of protospacers. During target recognition, Cascade-crRNA first recognizes the PAM and then, if there is complementarity between the crRNA spacer and the target strand of protospacer, forms an R-loop complex: the target strand of protospacer forms a heteroduplex with crRNA spacer, the nontarget strand is looped out [14,15,16,17]. The R-loop complex recruits Cas3 [15,18,19]. In vitro, Cas3 introduces several nicks into the nontarget strand 7-11 nucleotides downstream from PAM. Cas3 possess a 3’-5’ helicase activity and in the presence of ATP is able to translocate along the target DNA, gradually degrading it to short fragments [17,20,21]. In vivo, Cas3 activity leads to extensive degradation of DNA around the protospacer [8,22], a process that may involve host nucleases and helicases that are not part of the CRISPR-Cas system.

CRISPR adaptation in *E. coli* is catalyzed by the Cas1_4_-Cas2_2_ hexameric complex [23,24,25]. Up to 50% of spacers acquired in cells co-overexpressing Cas1 and Cas2 correspond to prespacers with adjacent AAG trinucleotides [13], which matches the PAM consensus. For type I-E CRISPR-Cas systems it was shown that ongoing interference drives a specific and very efficient mode of CRISPR adaptation, referred to as “primed CRISPR adaptation” or “priming” [26,27]. In this case, up to 95% of newly acquired spacers correspond to prespacers associated with AAG PAM, and most of them originate from an area around the protospacer recognized by Cascade-crRNA (referred to as “*p*riming *p*roto*s*pacer” or “PPS”) [28]. Further, the orientation of priming protospacer affects the choice of acquired spacers. It has been proposed that the products of target degradation initiated by Cas3 feed spacer acquisition during primed adaptation [29]. 

The detailed mechanism of conversion of DNA in a locus targeted by Cascade-crRNA and Cas3 to spacers in CRISPR array during primed CRISPR adaptation remains to be identified. It is highly likely that besides the Cas3 helicase-nuclease and the Cas1-Cas2 adaptation complex, cellular machinery engaged in genome maintenance and DNA metabolism also contributes to primed spacer acquisition. Indeed, the requirement of *recG* and *priA* for primed CRISPR adaptation was demonstrated using a lambda phage infection model [30]. Here, we analyzed the role of genome maintenance components – the RecBCD and SbcCD complexes as well as the RecJ and SbcB nucleases—in spacer acquisition efficiency and specificity during primed adaptation using the “self-targeting” *E. coli* strain recently described in [31]. We found that primed adaptation is strongly affected by RecBCD, SbcD, and RecJ, which thus should be considered as integral players of the process.

## 2. Materials and Methods

### 2.1. Strains Used in This Study

**Figure 1:**
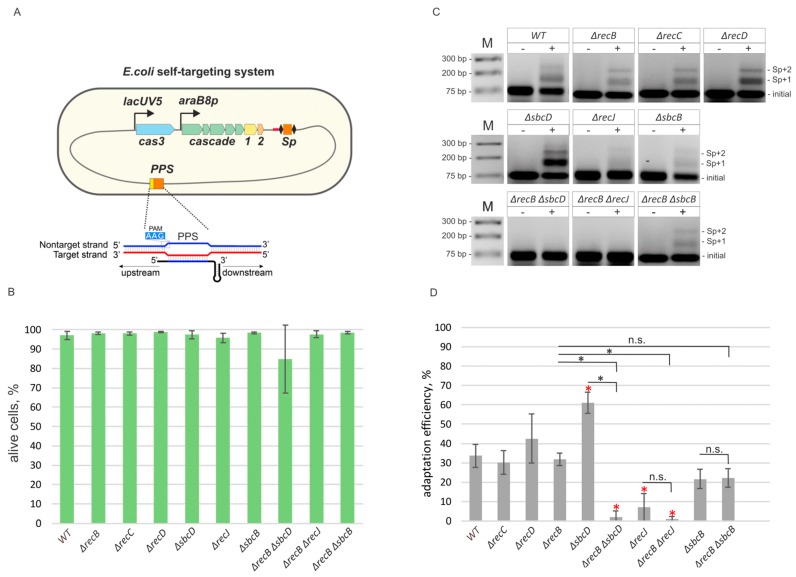
Components of genome maintenance pathways contribute to primed adaptation efficiency. (**A**) A self-targeting *E. coli* strain KD403 used to study the effects of non-CRISPR genes of primed adaptation is schematically shown. KD403 contains the *cas3* gene under IPTG-inducible *lacUV5* promoter and the *cse1*, *cse2*, *cas5*, *cas6*, *cas7*, *cas1*, *cas2* operon under the control of arabinose-inducible promoter *araB8p*. A minimized CRISPR array contains a single spacer (Sp) corresponding to a priming protospacer (PPS) in the non-essential *yihN* gene. The leader region is shown in red. The structure of the R-loop complex on PPS is shown in the inset. (**B**) Cultures of KD403 self-targeting cells and derivatives with mutations in *recB*, *recC*, *recD*, *sbcD*, *recJ*, *sbcB*, *recB sbcD*, *recB recJ*, and *recB sbcB* genes were grown in the presence (“+”) or the absence (“−“) of inducers and the number of live cells at 5 h post induction was measured by live fluorescence microscopy. (**C**) Spacer acquisition determined by PCR amplification of the CRISPR array in uninduced (“−“) and induced (“+“) cells 5 h post-induction. Amplification products were resolved by agarose gel electrophoresis. Amplicons corresponding to initial, unexpanded CRISPR array and arrays expanded by one (“Sp+1”) and two (“Sp+2”) spacers-repeat units are indicated. A representative result of one of three independent experiments is shown. Data for two additional independent experiments (along with the one shown here) are presented in Figure S5. (**D**) Adaptation efficiency was measured as percentage of intensities of upper bands, corresponding to extended arrays, of the sum of intensities of upper and lower bands of agarose gels shown in panel **C** and normalized to values obtained for samples grown in the “absence” of inducers. The mean and standard deviations for three independent experiments are shown. Samples that significantly differ from wild-type KD403 strain are marked by red asterisks (pairwise t-test, *p*-value < 0.01). The brackets above the bars indicate pairwise t-test comparisons between double mutants and corresponding single mutants (n.s., non-significant differences at *p*-value ≥0.01; black asterisks, significant differences at *p*-value < 0.01).

The KD403 strain was described in [31] and also shown in Figure 1A. P1 transduction was used to replace *recB*, *recC*, *recD*, *sbcB*, *sbcD*, *recJ* genes in the strain KD403 with the cassette encoding the gene of kanamycin resistance as described previously [32]. Keio collection strains [33] were used as donor strains. To obtain double mutants, the kanamycin cassette was flipped out from single knockouts using FLP recombinase expressed from pCP20 plasmid as described [34], and then the second round of P1 transduction was applied to introduce the second deletion.

### 2.2. Primed Adaptation Assay in “Self-Targeting” Cells

Cells were grown overnight at 37 °C in Luria-Bertani (LB) broth. Aliquots of the cultures were diluted 100-fold into fresh 20 mL of LB broth and grown at 30 °C. After reaching OD_600_ 0.3, the cultures were divided into two aliquots (7 mL each), and IPTG (isopropyl β-D-1 thiogalactopyranoside) and L-arabinose were added to one of two aliquots at the final concentration 1 mM each. The cultures were grown at 30 °C for 5 h, washed with 10 mL of 1x PBS buffer, resuspended in 700 μL of 1× PBS buffer and stored at −20 °C. Genomic DNA was purified from cultures by phenol-chloroform extraction as described in [31]. CRISPR expansion was monitored by PCR with primers annealing to Sp*^yihN^* spacer (auto- Sp*^yihN^* -R, 5′- aatagcgaacaacaaggtcggttg-3′) and the leader of the CRISPR array (LDR-F2, 5′- atgctttaagaacaaatgtatacttttag-3′). ImageJ [35] was used for quantitative analysis of adaptation efficiency. To measure the relative intensities of the bands on agarose gel corresponding to extended CRISPR arrays, the intensities in rectangles that cover corresponding areas were divided by sum of intensities of the same size areas of upper (extended) and lower (unextended) bands and normalized to the values calculated for samples without induction. Three independent experiments were done for each strain. Pairwise t-test with Holm correction for multiple comparisons was used for statistical analysis of differences in adaptation efficiency between different strains.

For extraction of CRISPR arrays expanded during CRISPR adaptation three independent 100 μL amplification reactions with LDR-F2 and auto- Sp*^yihN^*-R primers containing 20–50 ng genomic DNA were pooled, PCR products corresponding to expanded CRISPR array were gel purified using GeneJET Gel Extraction Kit (Thermo Fisher Scientific, Vilnius, Lithuania)) and sequenced in pair-end mode with MiniSeq Illumina System at Skoltech Genomics Core facility. Two independent experiments were analyzed by Illumina sequencing for each strain.

### 2.3. Microscopy

Cells of cultures grown with or without induction of *cas* gene expression were analyzed using a LIVE/DEAD bacterial viability kit (Thermo Scientific) at 5 hr after induction in cell chambers made as described in [31]. Fluorescence microscopy was performed using Nikon Eclipse Ti-E inverted microscope. Fluorescence signals in green (living cells) and red (dead cells) fluorescent channels were detected using Semrock filter sets YFP-2427B and TxRed-4040C, respectively, and numbers of corresponding cells were calculated. The image analysis was performed using ImageJ (Fiji) with ObjectJ plugin used for measurements of cell length.

### 2.4. Sequencing Data Processing

Raw sequencing data were processed using ShortRead and BioStrings R packages [36,37]. Illumina reads were trimmed with quality score cutoff = 20. Paired reads were merged with the following parameters: gap opening = −10, gap extension = −4. Reads containing two or more repeats (with up to two mismatches) were selected. The 33-bp segments were considered as spacers and were mapped to the KD403 genome sequence with no mismatches and only unique mapping allowed. A minor fraction of non-uniquely mapped spacers (i.e., those originating from rRNA operons) was excluded from the analysis. Spacers that have been inaccurately incorporated, i.e., those shifted by a few nucleotides with respect to the consensus PAM or inserted in an opposite orientation [38] were also discarded from the analysis except when inaccurately incorporated spacers were specifically analyzed (see below). Custom R scripts used for spacer analysis are available upon request. Spacers were counted in bins of 10 kb separately for target and nontarget strands and normalized to the total spacer counts. For plotting prespacer distribution, we averaged results across two independent experiments, also, to directly compare the curves, maximal values for mutants and wild-type were adjusted to make them the same. A portion of spacers associated with AAG PAM as well as a portion of spacers with internal AAG trinucleotide was calculated for 400 kb region spanning the PPS area in bins of 10 kb separately for nontarget and target strands. Spacers that mapped next to prespacers associated with AAG PAM but shifted up to 2 bp up- or downstream, and/or inserted in an opposite orientation, were considered as incorrectly incorporated. Their numbers were also calculated in 10 kb bins in the 400 kb PPS-centered area. A pairwise proportional test that accounts for multiple testing at p-value of 0.001 was applied to compare the values obtained for mutant strains with corresponding values for the wild-type strain. 

## 3. Results

### 3.1. Primed CRISPR Adaptation Is Impaired in ΔrecJ, ΔrecB ΔrecJ and ΔrecB ΔsbcD Mutants

Recently, a new in vivo model system to study primed adaptation at conditions of self-targeting in *E. coli* has been thoroughly described (see for details [31]). It is based on a strain KD403, which contains a CRISPR array spacer targeting a non-essential *yihN* gene in the *E. coli* genome, and *cas* genes responsible for CRISPR interference and adaptation under control of inducible promoters (Figure 1A). Upon *cas* genes induction, the protospacer in the *yihN* gene is recognized, and extensive DNA degradation at both sides of the protospacer is observed in a process that requires Cas3 nuclease activity [31]. The DNA degradation causes SOS response: the cultures stop growing, the number of colonies forming units in the culture is decreased several orders of magnitude (Figures S1 and S2). Induced cells become elongated but remain viable for at least several hours post-induction ([31] and Figure 1B, Figures S3 and S4) and acquire new spacers. Most of the newly acquired spacers originate from the *yihN* protospacer (priming protospacer, PPS) area [31]. 

To find out how cell nucleases and helicases involved in genome maintenance and repair affect spacer acquisition at conditions of self-targeting, we first evaluated the role of the RecBCD complex, which is involved in homologous recombination induced by double-strand DNA breaks. After binding to the ends of a broken DNA duplex, RecBCD translocates along the DNA using 3’-5’ and 5’-3’ helicase activities of the RecB and RecD subunits, correspondingly, until a *Chi* site is recognized by the RecC subunit [39,40]. Whether RecBCD degrades DNA as it is moving along in vivo is still a subject to debate [39,41]. In vitro RecBCD possesses a Mg^2+^-dependent nuclease activity [42]. Upon *Chi* site recognition, RecB turns into an efficient processive 5’-3’ nuclease producing 3’ overhangs that initiate the process of homologous recombination. The absence of RecD converts the RecBC complex into a helicase that lacks the nuclease activity [43]. Previously, the role of the RecBCD complex in primed adaptation was assessed, and no impact was found [30]. We used P1 transduction to generate derivatives of the KD403 self-targeting strain with *recB*, *recC*, or *recD* genes replaced by kanamycin resistance cassette (see Materials and Methods). As in the parental, wild-type KD403 strain, induction of self-targeting in the mutants caused SOS response, as judged by the increase of cell length (Figures S3 and S4), cessation of culture growth (Figure S1), and reduction in colony forming units (Figure S2) but did not increase the number of dead cells 5 h after the addition of *cas* genes inducers as judged by differential staining of live and dead cells (Figure 1B and Figure S3). Spacer acquisition, detected by the appearance of PCR amplicons corresponding to extended arrays 5 h post-induction, was observed at a level comparable to that observed in the parental KD403 (Figure 1C,D, Figure S5), suggesting that neither RecBC nor RecBCD are required for primed adaptation.

By constructing appropriate KD403 derivatives, we next assessed the role of 3’–5’ single-strand specific exonuclease SbcB, 5’–3’ single-strand specific exonuclease RecJ, and of SbcD, a component of a single- and double-stranded DNA 3’–5’ exonuclease and endonuclease SbcCD. The SbcB protein, also known as Exonuclease I, interacts with SSB (single-strand DNA binding protein) [44]. By performing highly processive 3’ end trimming it participates in gap repair and blunting of DNA ends to allow RecBCD binding [45,46]. RecJ is a processive 5’–3’ exonuclease involved in excision repair and homologous recombination [47]. The SbcCD complex recognizes and cleaves hairpin structures formed by palindromic sequences in vitro [48] and in vivo [49]. It was reported to possess dsDNA exonuclease and ssDNA 3’–5’ exonuclease activities [50]. Although a functional activity of these proteins in vivo remains elusive, they are thought to be involved in DNA processing at the sites of double-stranded breaks and/or replication fork stalling, and may, therefore, interfere with spacer precursor selection and/or maturation during primed adaptation. RecJ and SbcD effects on primed adaptation in the phage infection model were considered insignificant in earlier studies [30]. Induction of self-targeting in *ΔsbcB*, *ΔsbcD*, and *ΔrecJ* mutants cultures led to increase of cell length (Figures S3 and S4), cessation of culture growth (Figure S1), and reduction in colony forming units (Figure S2) comparable to what was seen in parental KD403 and had either no effect on spacer acquisition (*ΔsbcB* mutant) or led to a ~2-fold (*ΔsbcD* mutant) increase or ~4-fold decrease (*ΔrecJ* mutant) in adaptation efficiency (Figure 1C,D).

Taking into consideration that some genes analyzed may encode enzymes with redundant activities, we assessed primed adaptation in double mutants *ΔrecB ΔrecJ*, *ΔrecB ΔsbcD,* and *ΔrecB ΔsbcB*. Induction of self-targeting in double mutants led to increase of cell length (Figures S3 and S4), cessation of culture growth (Figure S1), and reduction in colony forming units (Figure S2) at levels comparable to those seen in parental KD403 cell cultures. As judged by live microscopy, the viability of double mutants was either not or mildly affected (15% dead cells for *ΔrecJ ΔsbcD* strain 5 h post-induction (Figure 1B, Figure S3), these cells also formed minute colonies in the absence of inducers (Figure S2)), which allowed us to compare the adaptation efficiency. While adaptation was not affected in the Δ*recB* Δ*sbcB* double mutant, it was not detectable in the *ΔrecB ΔrecJ* and *ΔrecB ΔsbcD* mutants, suggesting that the presence of functional *cas3* and *cas1-cas2* alone is not sufficient for spacer acquisition at wild-type level in these cells (Figure 1C,D).

### 3.2. Deletions in recB, recC, recD, and sbcD Genes Affect the Choice of Spacers Acquired during Primed Adaptation

**Figure 2:**
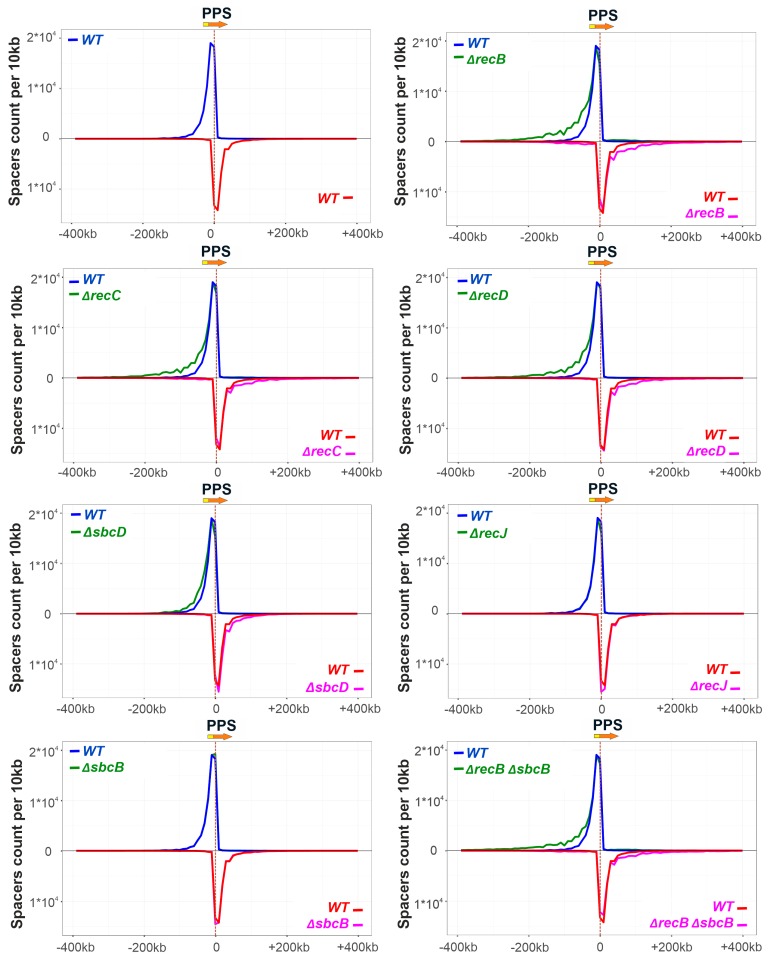
Components of genome maintenance pathways affect the extent of the area around the priming protospacer (PPS) from which spacers are selected. At the top left panel, unique spacers acquired by KD403 are mapped to the area of the genome 400 kbp upstream and downstream of the PPS. The orientation of PPS is shown by a red arrow at the top, with yellow fragment at the beginning of the arrow denoting protospacer adjacent motif (PAM). The numbers of spacers mapping to 10 kb bins at each side of the PPS are plotted in a strand-specific manner. A curve showing the distribution of spacers mapping to nontarget strand is colored blue and plotted above the X-axis; a curve showing the distribution of spacers mapping to the target strand is colored red and plotted below the X-axis. The rest of the panels show normalized comparisons of spacer acquisition profiles between the parental (“WT”) KD403 and indicated mutants where spacer acquisition was detected (see Figure 1). In each panel, the wild-type profile is shown by blue and red colored lines, while the mutant profiles are green (nontarget strand) and magenta (not-target strand). Averaged results for two biological replicates are shown. For *ΔrecJ* and *ΔsbcB* mutants the mapping curves are completely superimposed on the WT curves and are hardly seen.

**Figure 3:**
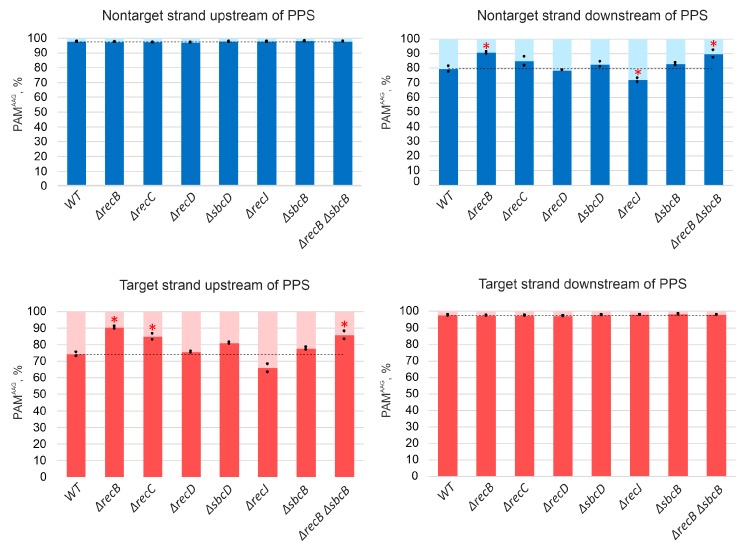
Association with the AAG PAM of spacers acquired by KD403 and its derivatives. Spacers acquired by indicated cells were mapped to an area within 200 kbp upstream and downstream of the PPS, and the percentage of mapping sites with an AAG PAM was determined separately for spacer mapping to upstream and downstream target and nontarget strands. The heights of bars show mean values obtained in two independent experiments (black dots show values obtained in each individual experiment). The dotted line shows the average level for the parental KD403 (“WT”) strain. Red asterisks mark values that significantly differ from the WT level according to pairwise proportional test with a *p*-value of 0.001.

To get a deeper insight into the role of genes under study in primed adaptation, amplicons corresponding to extended CRISPR arrays that acquired new spacers were analyzed by high-throughput sequencing (see Materials and Methods). Sequences from Illumina reads flanked by CRISPR repeats were treated as spacers, extracted, and mapped to the KD403 genome. It has been shown that in KD403 newly acquired spacers correspond to prespacers located in an area surrounding the PPS with a characteristic gradient of acquisition efficiency falling as the distance from the PPS increases [31]. Further, acquired spacers (taken as sequences of non-transcribed strand of the CRISPR array), preferentially map to the nontarget strand upstream of the PPS and the target strand downstream of it, with more than 96% of spacers originating from sequences with an AAG PAM. According to previously published data, 97% of spacers acquired by the KD403 cultures originate from a 400-kbp region upstream and downstream of the PPS. 59% of spacers correspond to prespacers located on the nontarget strand upstream of the PPS while 41% of spacers correspond to prespacers located on the target strand downstream of it [22,31] (Figure 2). 98% of these “strand-biased” spacers originate from sequences associated with an AAG consensus PAM, a hallmark of primed adaptation [26] (Figure 3).

No spacers acquired due to priming at PPS were detected in DNA amplified from *ΔrecB ΔrecJ* and *ΔrecB ΔsbcD* double mutants, in agreement with lack of detectable expanded arrays bands in Figure 1D, so they were excluded from further analysis. Though the overall yield of spacer acquisition in *ΔrecB*, *ΔrecC*, and *ΔrecD* mutants, as judged by PCR analysis of CRISPR array amplicons, was the same as in parental KD403, mapping of acquired spacers revealed a clear alteration in their pattern. Specifically, compared to the “wild-type” strain, the area, from which spacers were acquired in the mutants was extended further away from the PPS in both directions. A similar, though less pronounced trend was observed for spacers acquired by the *ΔsbcD* mutant (Figure 2). In contrast, compared to parental KD403, no changes were observed in the size of the area from which spacer acquisition occurred in *ΔrecJ* or *ΔsbcB* single mutants or in the *ΔrecB ΔsbcB* double mutant compared to the *ΔrecB* single mutant.

For all strains analyzed, the average association with AAG for spacers mapping to “preferred” strands upstream and downstream of the PPS was at a level of at least 97% (Figure 3). This was also true for PPS-distal “extra” spacers acquired in *ΔrecB*, *ΔrecC, ΔrecD,* and *ΔsbcD* mutants from regions that were not functioning as spacer donors in parental KD403 (Figure S6).

A minor (~3%) fraction of spacers acquired by induced KD403 cultures was mapped to the target strand upstream of the PPS and to the nontarget strand downstream of it. The acquisition of these spacers is clearly driven by the PPS recognition, as they cluster in the area around the PPS and in this sense originate through priming. Interestingly, only 79% and 74% of these spacers originate from sequences associated with the AAG PAM (Figure 3). These values, while clearly higher than those reported for naïve adaptation (40–50%) [51], are considerably below the values typical for primed adaptation, suggesting that a mechanistically distinct process is involved in prespacer selection. Yet, the area of acquisition of minor spacers mapping to opposite strands was extended in *ΔrecB*, *ΔrecC, ΔrecD,* and *ΔsbcD* mutants and unaffected by deletions of *recJ* or *sbcB*, similar to the situation observed with spacers acquired during the *bona fide* primed adaptation process. Surprisingly, when analyzing the AAG bias of minor spacers acquired in different KD403 mutant derivatives, we consistently observed significantly more AAG-associated spacers in the *ΔrecB* mutant but not in the *ΔrecD* mutant (Figure 3). Although the detailed mechanism remains to be established, our observation highlights the distinct roles of helicase and nuclease activities of RecBCD in primed adaptation.

### 3.3. Deletion of recJ Influences Prespacer Integration

**Figure 4:**
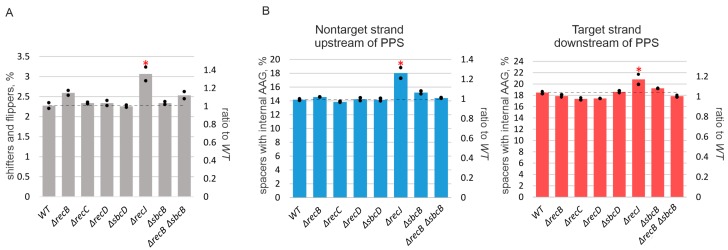
RecJ affects spacer acquisition precision and specificity. Spacers acquired by indicated cells were mapped to an area within 200 kbp upstream and downstream of the PPS for KD403 and indicated derivatives. (**A**) Percentage of inaccurately incorporated spacers (both shifters and flippers [38]) in indicated cultures. The numbers on the right axis show the ratios of inaccurately incorporated spacers to those obtained for *WT* cells. (**B**) Percentage of spacers with internal AAG trinucleotides. Data for spacers mapping to nontarget strand upstream of the PPS and target strand downstream are shown by blue and red bars, correspondingly. The heights of bars show mean values obtained in two independent experiments (black dots show values obtained in each individual experiment). The numbers on the right axis show the ratios of spacers with internal AAG trinucleotides to those obtained for *WT* cells. The dotted line shows the average level for the parental KD403 (“WT”) strain. Red asterisks mark values that significantly differ from the WT level according to pairwise proportional test with a *p*-value of 0.001.

The deletion of *recJ* decreased primed adaptation efficiency ~4-fold (Figure 1C,D). Simultaneous deletion of *recJ* and *recB* made primed adaptation undetectable (Figure 1C,D). Since in contrast to *ΔrecBCD* mutants the absence of *recJ* did not shift the area from which spacers came from (Figure 2), we assumed that the pathways that involve RecBCD and RecJ in primed adaptation are non-redundant. In other words, each pathway could make its own, independent, contribution, and abolishing both pathways prevents primed adaptation. To get further insights into the role of RecJ, we investigated the repertoire of acquired spacers in the *ΔrecJ* mutant. Spacers were clearly acquired less accurately in the absence of RecJ than in all other strains (Figure 4A). The percentage of inaccurately incorporated spacers that have been shifted relative to the AAG PAM in prespacers or inserted into the CRISPR array in the opposite orientation was 2.25% of the total acquired spacers in parental KD403 and *ΔrecBCD* and *ΔsbcD* mutants, but was 3% in the *ΔrecJ* mutant (a 30%, highly statistically significant increase from the wild-type level). Second, the choice of acquired spacers was affected in the *ΔrecJ* mutant in a very specific way. Recently, it was shown that spacers efficiently acquired in the course of primed adaptation are depleted of internal AAG trinucleotides [52]. The percentage of spacers containing internal AAG trinucleotide was slightly, yet, significantly increased in the *ΔrecJ* mutant (Figure 4B). Consistently, compared to parental KD403, the AAG PAM bias was slightly decreased (though at the border of statistical significance) for acquired spacers mapping to poorly used strands around the PPS in *ΔrecJ* cells (Figure 3).

## 4. Discussion

Naïve and primed CRISPR adaptation involve generation of prespacers and their incorporation into the CRISPR array. In naïve CRISPR adaptation, the efficiency of prespacer selection varies for different genomic regions and is stimulated by the breaks in genomic DNA [53]. No preferences in selection of prespacers with specific orientation of the PAM sequence have been reported so far. The stark difference of primed adaptation is that newly acquired spacers mostly target protospacers located upstream of the PPS and flanked by the PAM sequence in the same orientation as for the PPS (AAG sequence in the nontarget strand; CTT sequence in the target strand) [26,27,28]. While prespacer integration performed solely by the Cas1-Cas2 complex should be similar for both naïve and primed adaptation, the mechanism responsible for prespacer generation must be the source of the differences observed.

The RecBCD complex affects naïve CRISPR adaptation, which proceeds in the absence of Cascade and Cas3, presumably by providing substrates for spacer acquisition [53]. The helicase but not the nuclease activity of RecBCD is required for naïve adaptation and it was suggested that Cas1-Cas2 is targeted to DNA end structures [54]. For primed adaptation, it was proposed that during CRISPR interference Cas3 cleaves target DNA into fragments used by Cas1-Cas2 as spacer precursors [27]. If that were the case, generation of Cas3-dependent fragments associated with either AAG or CTT sequence depending on a strand would be expected. In vitro, Cas3 cleaves a PPS-containing plasmid bound by a Cascade-crRNA complex into double-stranded fragments that can be bound by Cas1-Cas2, processed and integrated into the CRISPR-array [29]. The Cas3-generated fragments are enriched with T in their 3’ ends that might contribute to a higher number of fragments containing the 3’-end CTT sequence [29]. However, the in vitro system does not recuperate the orientation bias of PAM sequences observed in vivo [26,27,28] since the enrichment with T in the 3’ ends was detected for both strands. Moreover, recently we have demonstrated that in vivo prespacers’ ends are enriched with CTTNN rather than CTT sequences [31]. This discrepancy indicates that the mechanism of primed adaptation in vivo might be much more complex than the simple cleavage of both strands by Cas3 followed by binding of these fragments by Cas1-Cas2. 

Due to degradation of the target DNA, host DNA repair and recombination proteins, such as RecBCD, SbcB, SbcCD and RecJ, can be recruited to the ends of the broken DNA in the PPS-region and these proteins might affect CRISPR interference and primed adaptation. The role of RecBCD in primed adaptation was not addressed but the RecG and PriA proteins were shown to be important in a model system targeting a phage genome [30]. In the present work, we address the effect of genome maintenance proteins on primed adaptation that occurs when CRISPR-Cas system targets host chromosome, a situation that has been observed in natural settings [55]. A critical advantage of self-targeting model system is that it allows one to avoid complications of popular systems that follow spacer acquisition from plasmids, for plasmids become unstable in Rec mutants, which severely affects the yields of acquired spacers [56,57,58]. We show that single deletion of *recJ* or simultaneous deletion of *recB* and *recJ* or *recB* and *sbcD* either strongly diminish primed adaptation or make it undetectable. Moreover, the pattern of acquired spacers was affected in the absence of RecBCD, SbcCD, or RecJ. Thus, at least at conditions of self-targeting, primed adaptation is not an autonomous process and depends on non-CRISPR machinery of the cell. 

**Figure 5:**
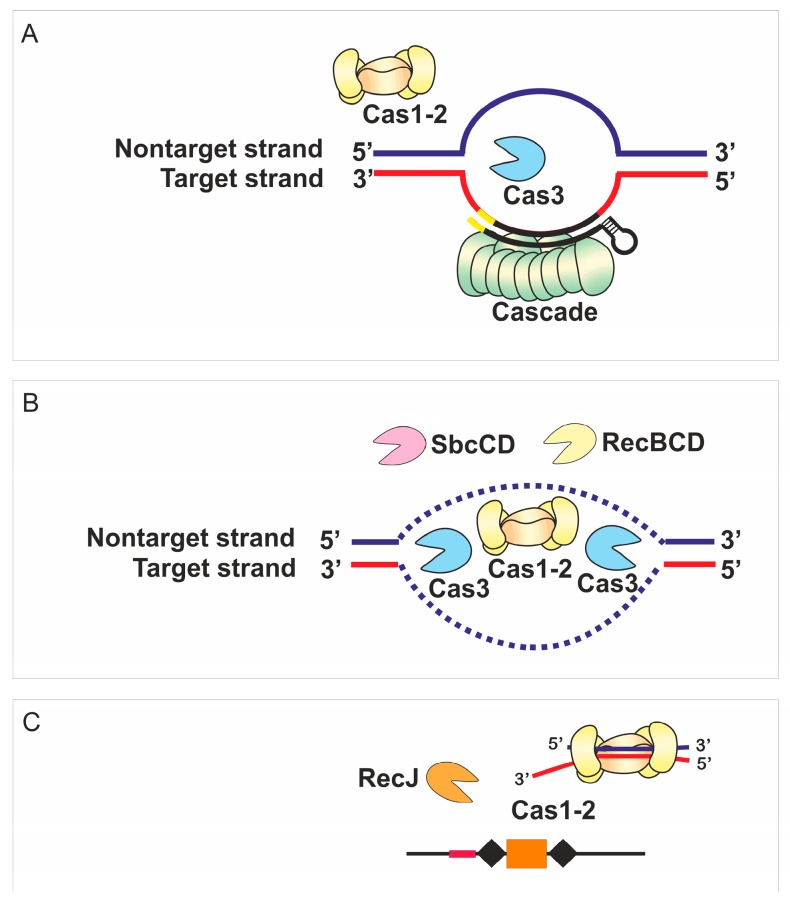
A possible mechanism of primed adaptation by the *E. coli* type I-E CRISPR-Cas system at conditions of self-targeting. (**A**) Target recognition and R-loop formation by the Cascade complex recruits the Cas3 nuclease-helicase required for CRISPR interference/target degradation. (**B**) The Cas1-Cas2 adaptation complex uses the products of DNA degradation to generate prespacers. Additional protein complexes (RecBCD, SbcCD) affect the pool of DNA intermediates available for Cas1-Cas2. (**C**) The Cas1-Cas2 adaptation complex interacts with prespacer fragments and channels them for insertion into the CRISPR array. RecJ finetunes the AAG specificity and precision of the adaptation complex at a later stage.

Both, direct and indirect, mechanisms of DNA maintenance machinery involvement in CRISPR adaptation may be at play as is shown in a schematic model presented in Figure 5. None of the proteins studied here affect strand specificity or high preference for AAG PAM of spacers originated from prespacers located on the nontarget strand upstream of the PPS and on the target strand downstream of it. This suggests that Cas3 recruited to PPS by the bound Cascade along with the Cas1-Cas2 adaptation complex are the main players, which determine characteristic features of primed spacer acquisition [29,59]. Single-molecule experiments with the purified Cas proteins from *Thermobifida fusca* demonstrated that Cas1, Cas2, Cas3 and Cascade form a primed acquisition complex (PAC) that translocates along DNA [59]. The assembly of PAC was also shown in vivo using a bimolecular fluorescence complementation assay [59]. The structure of this complex is not known but it is tempting to assume that the arrangement of the PAC components enables recognition of the AAG PAM in only one strand. Once the Cas1-Cas2 complex binds to the prespacer, further processing of protruding DNA ends may be required to form a 33-bp prespacer with a 3’-end overhang on a PAM-derived end [31]. In the present work we demonstrate that the simultaneous deletion of *recB* and *sbcD* makes spacer acquisition undetectable. Yet, removal of RecBCD or SbcCD alone has no such effect suggesting that activities of the RecBCD and SbcCD complexes in primed adaptation are redundant. Taking into account the nuclease activities of RecBCD and SbcCD complexes, these complexes might be involved in prespacer trimming.

Analysis of acquired spacers patterns in single mutants suggests that in the wild-type cells the RecBCD and SbcCD complexes interfere with the processivity of the primed adaptation complex as it moves away from the PPS. As a result, the genomic region around the PPS, from which spacers originate, is expanded in the *ΔsbcD*, *ΔrecB, ΔrecC* and *ΔrecD* mutants. RecBCD is a powerful motor translocating along dsDNA at velocity ~1.5 kbp/s and evicting protein roadblocks [60,61]. For comparison, several studies reported different values of Cas3 mean velocity but it did not exceed 500 bp/s [59,62,63]. It may be assumed that while Cas3 infrequently cuts DNA, the RecBCD complex binds to the broken DNA end and moves along the duplex until it reaches the Cas3 protein due to a higher speed of the RecBCD resulting in displacement of Cas3 from DNA. SbcCD is less studied but its ability to remove proteins from DNA was reported [64]. 

A minor portion of spacers originates from the target strand upstream of the PPS and the nontarget strand downstream of it. These spacers have decreased preference for AAG PAM. We assume that RecBCD complex directly participates in the creation of precursors of such spacers, as its abrogation modulates their specificity to AAG PAM. 

Deletion of *recJ* strongly diminishes, while simultaneous deletion of *recJ* and *recB* makes adaptation undetectable. Interestingly, the absence of RecJ also affects precision of spacer incorporation, increasing the number of incorrectly incorporated spacers by 30%. In addition, compared to the wild-type, more spacers with internal AAG trinucleotides are acquired in the *ΔrecJ* mutant. These observations seem to suggest that the 5’–3’ exonuclease activity of RecJ affects spacer acquisition *after* RecBCD and SbcCD, contributing to the specificity of pre-spacer maturation and the accuracy of their insertion in the array. Although RecJ is not a strictly required component of primed CRISPR adaptation in the self-targeting model, its attenuation of both the efficiency and specificity of the process merits further studies. Several studies demonstrated that the ideal substrates for the Cas1-Cas2 complex are double-stranded fragments with 3’ overhangs [25,65]. It was reported that 3’ ends of prespacers are cleaved by Cas1 [24] or by DnaQ-like domain of Cas2 in the type I-E system of *Streptococcus thermophilus* [60]. How 5’ ends of prespacers are trimmed was never addressed. RecJ might be involved in this trimming due to its 5’–3’ exonuclease activity.

Bioinformatic surveys revealed both positive and negative correlations between the co-occurrence of CRISPR-Cas systems and certain components of DNA repair machinery in different bacterial phyla suggesting mechanistic interactions [66]. Specifically, for the type I-E CRISPR-Cas systems, co-occurrence with SbcB, SbcCD, and RecBCD complexes was observed. Earlier reports, as well as data presented here establish that at least for the latter two complexes there indeed exists a functional interplay with primed adaptation. The experimental strategy reported in this work shall allow to experimentally address this question and also to study the functional interplay between genomic maintenance mechanisms and CRISPR-Cas systems of different types in other organisms.

## Supplementary Materials

The following are available online at https://www.mdpi.com/2073-4425/10/11/872/s1, Figure S1: Growth curves showing O.D. 600 of indicated cultures of self-targeting cells in the absence (left) or in the presence (right) of cas gene inducers, Figure S2: Aliquots of serial dilutions of cultures of indicated self-targeting cells with (right) and without (left) inducers of cas gene expression were spotted on LB plates five hours post-induction, Figure S3: Induction of self-targeting induces SOS-response. The representative images of cells under induction of self-targeting along with their un-induced counterparts (see Materials and Methods), Figure S4: Aliquots of induced and uninduced cultures of indicated self-targeting cells collected five hours post-induction were subjected to live microscopy and the length of cells was determined, Figure S5: Results of CRISPR adaptation experiments in three independent experiments (“1”, “2”, and “3”) are shown. See Figure 1C legend for details, Figure S6: Association with the AAG PAM of spacers acquired by KD403 and its derivatives in an extended area around the PPS.

## References

[1] Barrangou R., Fremaux C., Deveau H., Richards M., Boyaval P., Moineau S., Romero D., Horvath P. (2007). CRISPRProvides Acquired Resistance Against Viruses in Prokaryotes. Science.

[2] Brouns S.J.J., Jore M., Lundgren M., Westra E., Slijkhuis R., Snijders A., Dickman M., Makarova K., Koonin E., van der Oost J. (2008). Small CRISPR RNAs Guide Antiviral Defense in Prokaryotes. Science.

[3] Marraffini L.A., Sontheimer E.J. (2008). CRISPR Interference Limits Horizontal Gene Transfer in Staphylococci by Targeting DNA. Science.

[4] Cañez C., Selle K., Goh Y.J., Barrangou R. (2019). Outcomes and characterization of chromosomal self-targeting by native CRISPR-Cas systems in Streptococcus thermophilus. FEMS Microbiol. Lett..

[5] Guan J., Wang W., Sun B. (2017). Chromosomal Targeting by the Type III-A CRISPR-Cas System Can Reshape Genomes in Staphylococcus aureus. mSphere.

[6] Vercoe R.B., Chang J.T., Dy R.L., Taylor C., Gristwood T., Clulow J.S., Richter C., Przybilski R., Pitman A.R., Fineran P.C. (2013). Cytotoxic Chromosomal Targeting by CRISPR/Cas Systems Can Reshape Bacterial Genomes and Expel or Remodel Pathogenicity Islands. PLoS Genet..

[7] Gomaa A.A., Klumpe H.E., Luo M.L., Selle K., Barrangou R., Beisel C.L. (2014). Programmable removal of bacterial strains by use of genome-targeting CRISPR-cas systems. MBio.

[8] Caliando B.J., Voigt C. (2015). A Targeted DNA degradation using a CRISPR device stably carried in the host genome. Nat. Commun..

[9] Marraffini L.A., Sontheimer E.J. (2010). CRISPR interference: RNA-directed adaptive immunity in bacteria and archaea. Nat. Rev. Genet..

[10] Mojica F.J.M., Díez-Villaseñor C., García-Martínez J., Almendros C. (2009). Short motif sequences determine the targets of the prokaryotic CRISPR defence system. Microbiology.

[11] Deveau H., Barrangou R., Garneau J.E., Labonté J., Fremaux C., Boyaval P., Romero D.A., Horvath P., Moineau S. (2008). Phage response to CRISPR-encoded resistance in Streptococcus thermophilus. J. Bacteriol..

[12] van der Oost J., Jore M.M., Westra E.R., Lundgren M., Brouns S.J.J. (2009). CRISPR-based adaptive and heritable immunity in prokaryotes. Trends Biochem. Sci..

[13] Yosef I., Goren M.G., Qimron U. (2012). Proteins and DNA elements essential for the CRISPR adaptation process in Escherichia coli. Nucleic Acids Res..

[14] Jore M.M., Lundgren M., van Duijn E., Bultema J.B., Westra E.R., Waghmare S.P., Wiedenheft B., Pul U., Wurm R., Wagner R. (2011). Structural basis for CRISPR RNA-guided DNA recognition by Cascade. Nat. Struct. Mol. Biol..

[15] Westra E.R., van Erp P.B.G., Künne T., Wong S.P., Staals R.H.J., Seegers C.L.C., Bollen S., Jore M.M., Semenova E., Severinov K. (2012). CRISPR Immunity Relies on the Consecutive Binding and Degradation of Negatively Supercoiled Invader DNA by Cascade and Cas3. Mol. Cell.

[16] Mulepati S., Orr A., Bailey S. (2012). Crystal structure of the largest subunit of a bacterial RNA-guided immune complex and its role in DNA target binding. J. Biol. Chem..

[17] Hochstrasser M.L., Taylor D.W., Bhat P., Guegler C.K., Sternberg S.H., Nogales E., Doudna J.A. (2014). CasA mediates Cas3-catalyzed target degradation during CRISPR RNA-guided interference. Proc. Natl. Acad. Sci. USA.

[18] Sinkunas T., Gasiunas G., Fremaux C., Barrangou R., Horvath P., Siksnys V. (2011). Cas3 is a single-stranded DNA nuclease and ATP-dependent helicase in the CRISPR/Cas immune system. EMBO J..

[19] Sinkunas T., Gasiunas G., Waghmare S.P., Dickman M.J., Barrangou R., Horvath P., Siksnys V. (2013). In vitro reconstitution of Cascade-mediated CRISPR immunity in Streptococcus thermophilus. EMBO J..

[20] Mulepati S., Bailey S. (2013). In vitro reconstitution of an Escherichia coli RNA-guided immune system reveals unidirectional, ATP-dependent degradation of DNA Target. J. Biol. Chem..

[21] Xiao Y., Luo M., Hayes R.P., Kim J., Ng S., Ding F., Liao M., Ke A. (2017). Structure Basis for Directional R-loop Formation and Substrate Handover Mechanisms in Type I CRISPR-Cas System. Cell.

[22] Strotskaya A., Savitskaya E., Metlitskaya A., Morozova N., Datsenko K.A., Semenova E., Severinov K. (2017). The action of Escherichia coli CRISPR-Cas system on lytic bacteriophages with different lifestyles and development strategies. Nucleic Acids Res..

[23] Nuñez J.K., Kranzusch P.J., Noeske J., Wright A.V., Davies C.W., Doudna J.A. (2014). Cas1-Cas2 complex formation mediates spacer acquisition during CRISPR-Cas adaptive immunity. Nat. Struct. Mol. Biol..

[24] Wang J., Li J., Zhao H., Sheng G., Wang M., Yin M., Wang Y. (2015). Structural and Mechanistic Basis of PAM-Dependent Spacer Acquisition in CRISPR-Cas Systems. Cell.

[25] Nuñez J.K., Harrington L.B., Kranzusch P.J., Engelman A.N., Doudna J.A. (2015). Foreign DNA capture during CRISPR-Cas adaptive immunity. Nature.

[26] Datsenko K.A., Pougach K., Tikhonov A., Wanner B.L., Severinov K., Semenova E. (2012). Molecular memory of prior infections activates the CRISPR/Cas adaptive bacterial immunity system. Nat. Commun..

[27] Swarts D.C., Mosterd C., van Passel M.W.J., Brouns S.J.J. (2012). CRISPR interference directs strand specific spacer acquisition. PLoS ONE.

[28] Savitskaya E., Semenova E., Dedkov V., Metlitskaya A., Severinov K. (2013). High-throughput analysis of type I-E CRISPR/Cas spacer acquisition in E. coli. RNA Biol..

[29] Künne T., Kieper S.N., Bannenberg J.W., Vogel A.I.M., Miellet W.R., Klein M., Depken M., Suarez-Diez M., Brouns S.J.J. (2016). Cas3-Derived Target DNA Degradation Fragments Fuel Primed CRISPR Adaptation. Mol. Cell.

[30] Ivančić-Baće I., Cass S.D., Wearne S.J., Bolt E.L. (2015). Different genome stability proteins underpin primed and naïve adaptation in E. coli CRISPR-Cas immunity. Nucleic Acids Res..

[31] Shiriaeva A.A., Savitskaya E., Datsenko K.A., Vvedenskaya I.O., Fedorova I., Morozova N., Metlitskaya A., Sabantsev A., Nickels B.E., Severinov K. (2019). Detection of Spacer Precursors Formed In Vivo During Primed CRISPR Adaptation. bioRxiv.

[32] Moore S.D. (2011). Assembling new escherichia coli strains by transduction using phage P1. Methods Mol. Biol..

[33] Baba T., Ara T., Hasegawa M., Takai Y., Okumura Y., Baba M., Datsenko K.A., Tomita M., Wanner B.L., Mori H. (2006). Construction of Escherichia coli K-12 in-frame, single-gene knockout mutants: The Keio collection. Mol. Syst. Biol..

[34] Datsenko K.A., Wanner B.L. (2000). One-step inactivation of chromosomal genes in Escherichia coli K-12 using PCR products. Proc. Natl. Acad. Sci. USA.

[35] Rasband W. (2012). ImageJ.

[36] Morgan M., Anders S., Lawrence M., Aboyoun P., Pagès H., Gentleman R. (2009). ShortRead: A bioconductor package for input, quality assessment and exploration of high-throughput sequence data. Bioinformatics.

[37] Pages H., Gentleman R., Aboyoun P., Gentleman R., DebRoy S. (2008). Biostrings: Efficient Manipulation of Biological Strings. R Package Version 2.50.2. https://bioconductor.riken.jp/packages/3.1/bioc/html/Biostrings.html/.

[38] Shmakov S., Savitskaya E., Semenova E., Logacheva M.D., Datsenko K.A., Severinov K. (2014). Pervasive generation of oppositely oriented spacers during CRISPR adaptation. Nucleic Acids Res..

[39] Smith G.R. (2012). How RecBCD Enzyme and Chi Promote DNA Break Repair and Recombination: A Molecular Biologist’s View. Microbiol. Mol. Biol. Rev..

[40] Dillingham M.S., Kowalczykowski S.C. (2008). RecBCD Enzyme and the Repair of Double-Stranded DNA Breaks. Microbiol. Mol. Biol. Rev..

[41] Wiktor J., Van Der Does M., Büller L., Sherratt D.J., Dekker C. (2018). Direct observation of end resection by RecBCD during double-stranded DNA break repair in vivo. Nucleic Acids Res..

[42] Ponticelli A.S., Schultz D.W., Taylor A.F., Smith G.R. (1985). Chi-dependent DNA strand cleavage by RecBC enzyme. Cell.

[43] Palas K.M., Kushner S.R. (1990). Biochemical and physical characterization of exonuclease V from Escherichia coli. Comparison of the catalytic activities of the RecBC and RecBCD enzymes. J. Biol. Chem..

[44] Molineux I.J., Gefter M.L. (1975). Properties of the Escherichia coli DNA-binding (unwinding) protein interaction with nucleolytic enzymes and DNA. J. Mol. Biol..

[45] Thoms B., Wackernagel W. (1998). Interaction of RecBCD enzyme with DNA at double-strand breaks produced in UV-irradiated Escherichia coli: Requirement for DNA end processing. J. Bacteriol..

[46] Kushner S.R., Nagaishi H., Templin A., Clark A.J. (1971). Genetic Recombination in Escherichia coli: The Role of Exonuclease I. Proc. Natl. Acad. Sci. USA.

[47] Corrette-Bennett S.E., Lovett S.T. (1995). Enhancement of RecA strand-transfer activity by the RecJ exonuclease of Escherichia coli. J. Biol. Chem..

[48] Connelly J.C., Leach D.R.F. (1996). The sbcC and sbcD genes of Escherichia coli encode a nuclease involved in palindrome inviability and genetic recombination. Genes Cells.

[49] Eykelenboom J.K., Blackwood J.K., Okely E., Leach D.R.F. (2008). SbcCD Causes a Double-Strand Break at a DNA Palindrome in the Escherichia coli Chromosome. Mol. Cell.

[50] Connelly J.C., De Leau E.S., Leach D.R.F. (1999). DNA cleavage and degradation by the SbcCD protein complex from Escherichia coli. Nucleic Acids Res..

[51] Yosef I., Shitrit D., Goren M.G., Burstein D., Pupko T., Qimron U. (2013). DNA motifs determining the efficiency of adaptation into the Escherichia coli CRISPR array. Proc. Natl. Acad. Sci. USA.

[52] Musharova O., Vyhovskyi D., Medvedeva S., Guzina J., Zhitnyuk Y., Djordjevic M., Severinov K., Savitskaya E. (2018). Avoidance of Trinucleotide Corresponding to Consensus Protospacer Adjacent Motif Controls the Efficiency of Prespacer Selection during Primed Adaptation. MBio.

[53] Levy A., Goren M.G., Yosef I., Auster O., Manor M., Amitai G., Edgar R., Qimron U., Sorek R. (2015). CRISPR adaptation biases explain preference for acquisition of foreign DNA. Nature.

[54] Radovcic M., Killelea T., Savitskaya E., Wettstein L., Bolt E.L., Ivancic-Bace I. (2018). CRISPR-Cas adaptation in Escherichia coli requires RecBCD helicase but not nuclease activity, is independent of homologous recombination, and is antagonized by 5’ ssDNA exonucleases. Nucleic Acids Res..

[55] Shmakov S.A., Sitnik V., Makarova K.S., Wolf Y.I., Severinov K.V., Koonin E.V. (2017). The CRISPR spacer space is dominated by sequences from species-specific mobilomes. MBio.

[56] Biek D.P., Cohen S.N. (1986). Identification and characterization of recD, a gene affecting plasmid maintenance and recombination in Escherichia coli. J. Bacteriol..

[57] Silberstein Z., Cohen A. (1987). Synthesis of linear multimers of OriC and pBR322 derivatives in Escherichia coli K-12: Role of recombination and replication functions. J. Bacteriol..

[58] Cohen A., Clark A.J. (1986). Synthesis of linear plasmid multimers in Escherichia coli K-12. J. Bacteriol..

[59] Dillard K.E., Brown M.W., Johnson N.V., Xiao Y., Dolan A., Hernandez E., Dahlhauser S.D., Kim Y., Myler L.R., Anslyn E.V. (2018). Assembly and Translocation of a CRISPR-Cas Primed Acquisition Complex. Cell.

[60] Finkelstein I.J., Visnapuu M.L., Greene E.C. (2010). Single-molecule imaging reveals mechanisms of protein disruption by a DNA translocase. Nature.

[61] Terakawa T., Redding S., Silverstein T.D., Greene E.C. (2017). Sequential eviction of crowded nucleoprotein complexes by the exonuclease RecBCD molecular motor. Proc. Natl. Acad. Sci. USA.

[62] Redding S., Sternberg S.H., Marshall M., Gibb B., Bhat P., Guegler C.K., Wiedenheft B., Doudna J.A., Greene E.C. (2015). Surveillance and Processing of Foreign DNA by the Escherichia coli CRISPR-Cas System. Cell.

[63] Loeff L., Brouns S.J.J., Joo C. (2018). Repetitive DNA Reeling by the Cascade-Cas3 Complex in Nucleotide Unwinding Steps. Mol. Cell.

[64] Connelly J.C., De Leau E.S., Leach D.R.F. (2003). Nucleolytic processing of a protein-bound DNA end by the E. coli SbcCD (MR) complex. DNA Repair.

[65] Nuñez J.K., Lee A.S.Y., Engelman A., Doudna J.A. (2015). Integrase-mediated spacer acquisition during CRISPR-Cas adaptive immunity. Nature.

[66] Bernheim A., Bikard D., Touchon M., Rocha E.P.C. (2019). A matter of background: DNA repair pathways as a possible cause for the sparse distribution of CRISPR-Cas systems in bacteria. Philos. Trans. R. Soc. B Biol. Sci..

