# Supplementary material for: Genome Maintenance Proteins Modulate Autoimmunity Mediated Primed Adaptation by the Escherichia coli Type I-E CRISPR-Cas System
Source: Genes (Basel). 2019 Oct 31;10(11):872. doi: 10.3390/genes10110872 (PMC6896009; doi:10.3390/genes10110872)
Supplement: Supplementary file 1 [file genes-10-00872-s001.pdf]

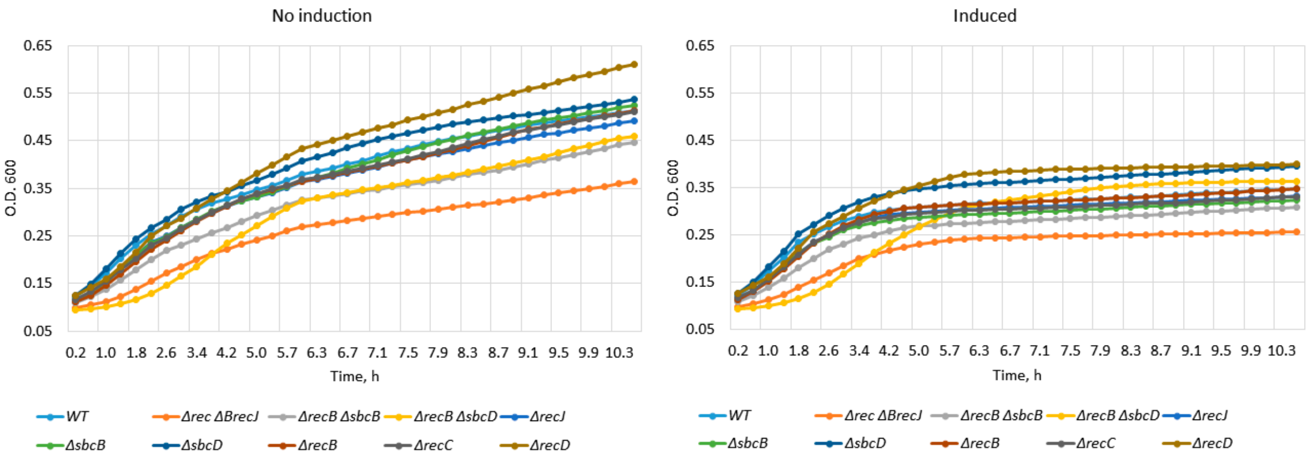

Figure S1. Growth curves showing O.D. 600 of indicated cultures of self-targeting cells in the absence (left) or in the presence (right) of *cas* gene inducers. Aliquots of cultures collected five hours post-induction were used for further analyses.

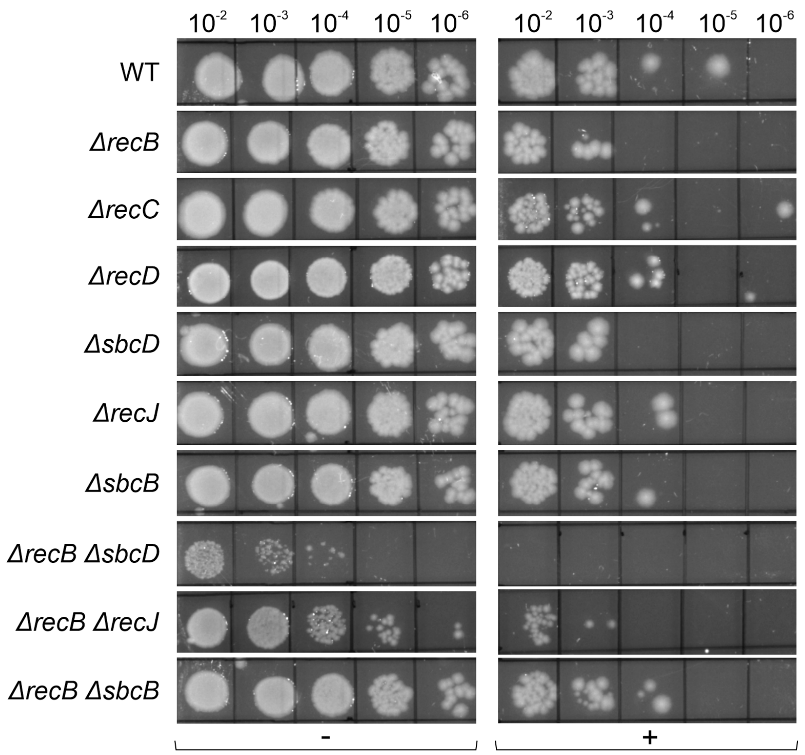

Figure S2. Aliquots of serial dilutions of cultures of indicated self-targeting cells with (right) and without (left) inducers of *cas* gene expression were spotted on LB plates five hours post-induction. The results of overnight growth at 37°C are presented.

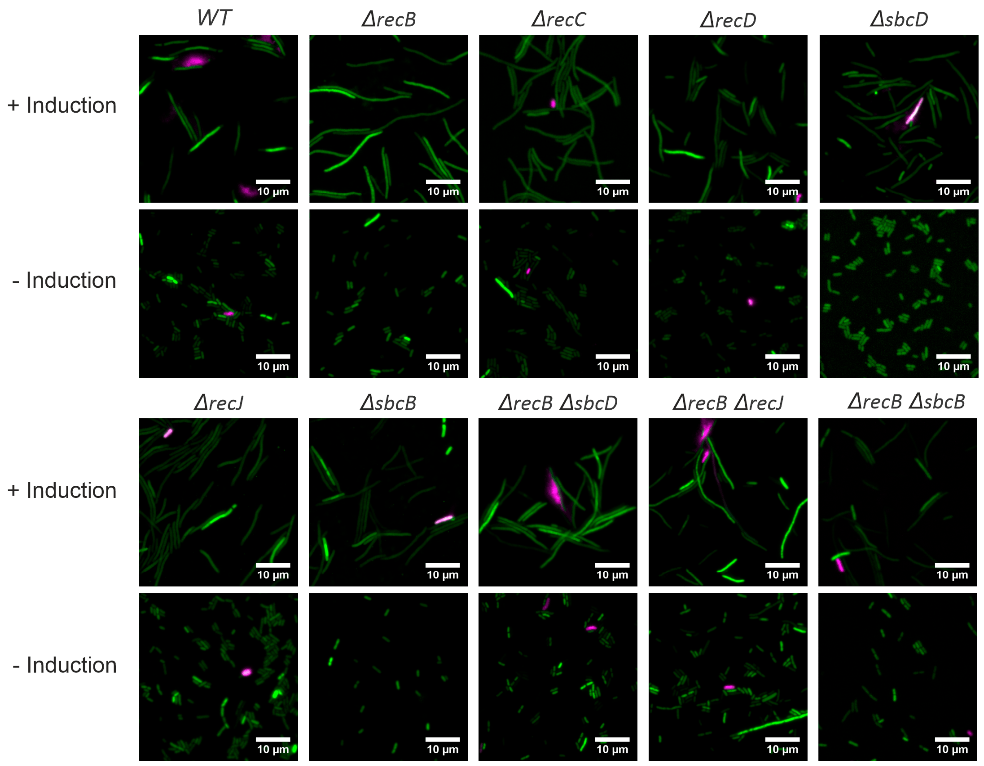

Figure S3. Induction of self-targeting induces SOS-response. The representative images of cells under induction of self-targeting along with their un-induced counterparts (see Materials and Methods). Red cells are dead ones, green cells are alive. Cells acquire elongated shape in response to double stranded breaks (SOS response).

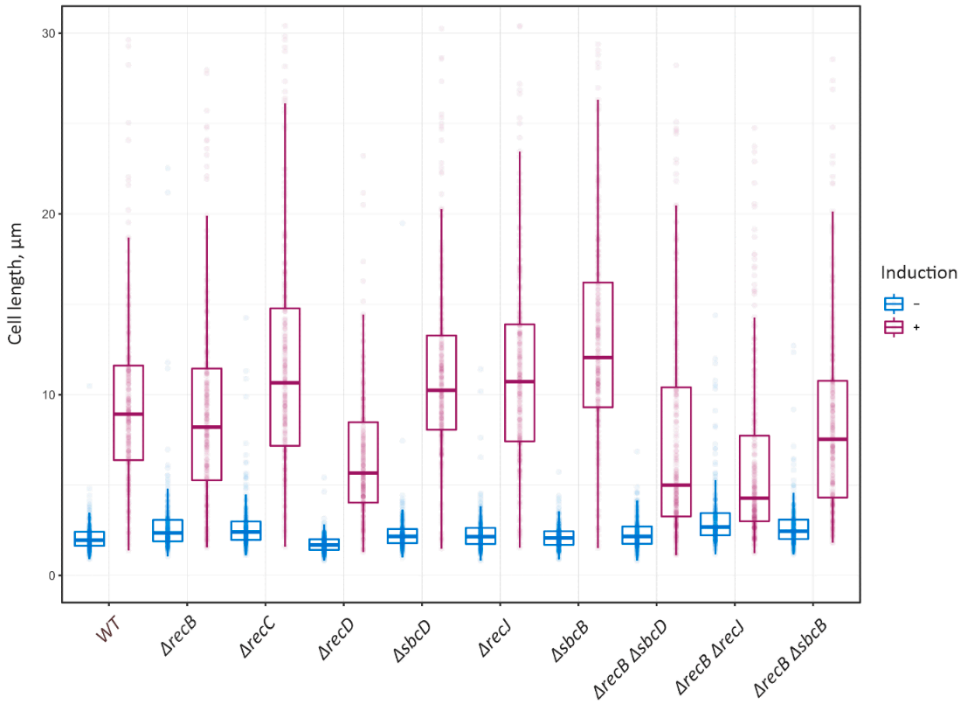

Figure S4. Aliquots of induced and uninduced cultures of indicated self-targeting cells collected five hours post-induction were subjected to live microscopy and the length of cells was determined. Boxplots showing the first quartile, the median, the third quartile and the whiskers extending to 1.5* IQR (inter-quartile range) are displayed for a total of 300 cells at each condition.

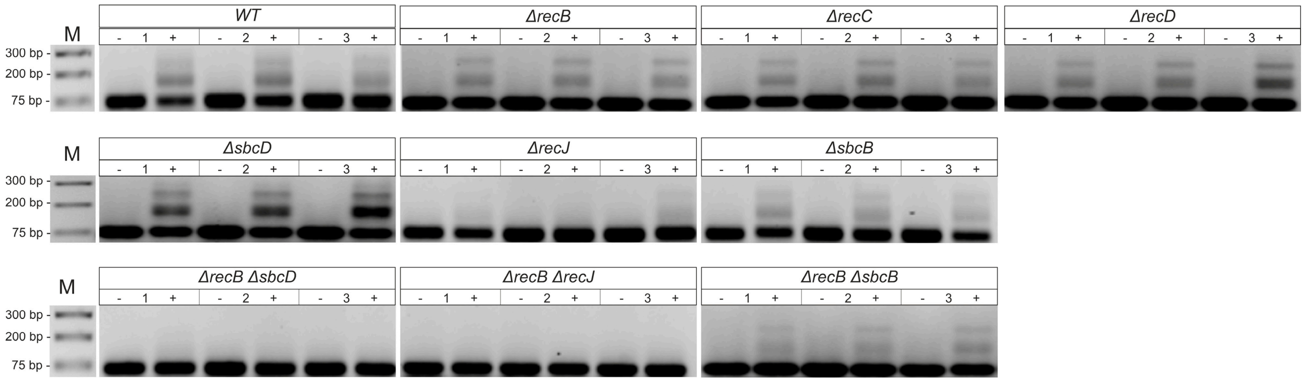

Figure S5. Results of CRISPR adaptation experiments in three independent experiments ("1", "2", and "3") are shown. See Figure 1C legend for details.

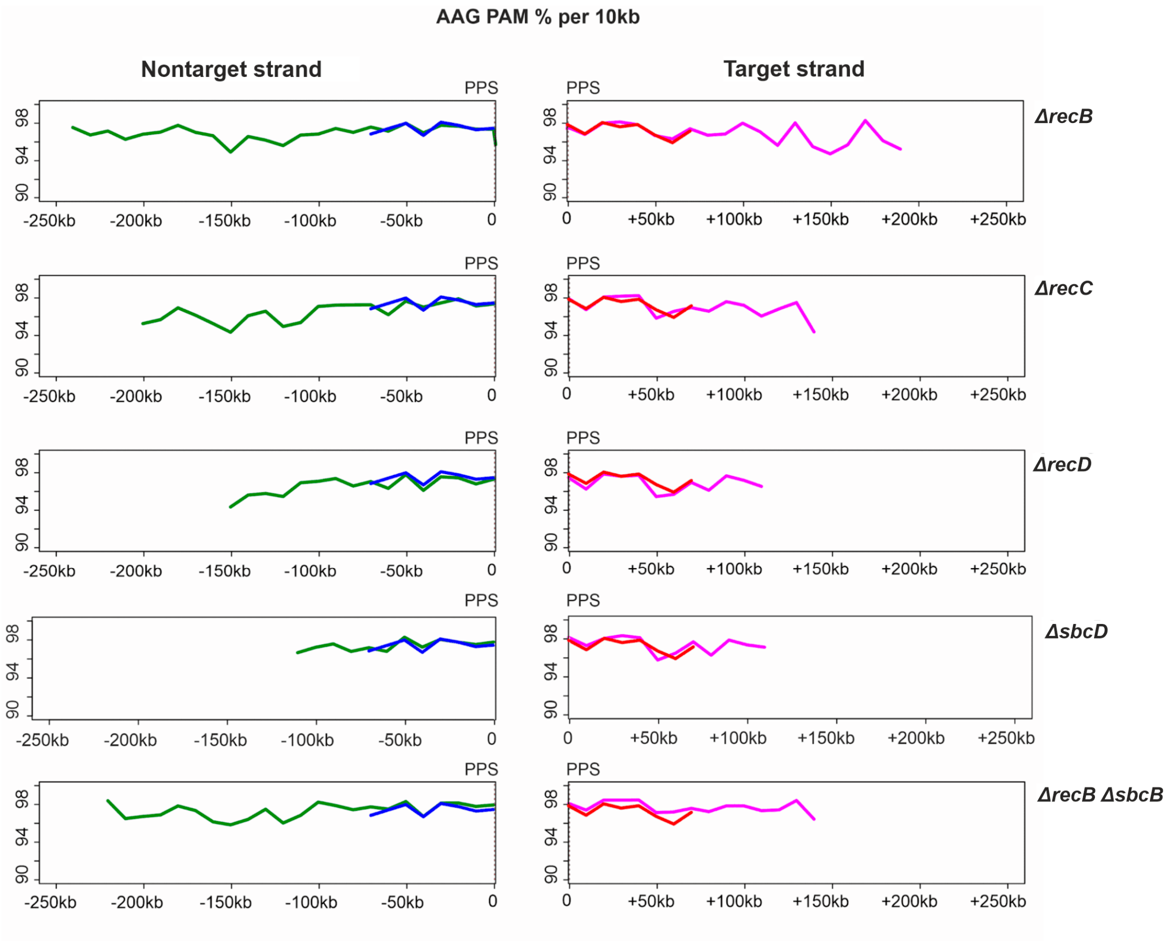

Figure S6. Association with the AAG PAM of spacers acquired by KD403 and its derivatives in an extended area around the PPS. Percentage of spacers mapping to sequences with appropriately positioned AAG PAM is plotted as a function of distance upstream and downstream of the PPS in 10 kb bins. Blue and red lines represent, correspondingly, values for nontarget strand upstream of the PPS and target strand downstream of the PPS for parental KD403. Green and purple lines represent the corresponding values for mutants. Only bins with number of spacers above a threshold of 500 counts were taken into consideration.
